# Alcohol Use, Screening, and Brief Intervention Among Pregnant Persons — 24 U.S. Jurisdictions, 2017 and 2019

**DOI:** 10.15585/mmwr.mm7203a2

**Published:** 2023-01-20

**Authors:** Jackie Luong, Amy Board, Lucas Gosdin, Janae Dunkley, JoAnn M. Thierry, Marc Pitasi, Shin Y. Kim

**Affiliations:** ^1^Division of Birth Defects and Infant Disorders, National Center on Birth Defects and Developmental Disabilities, CDC; ^2^Oak Ridge Institute for Science and Education, Oak Ridge, Tennessee; ^3^Division of Human Development and Disability, National Center on Birth Defects and Developmental Disabilities, CDC; ^4^Division of HIV Prevention, National Center for HIV, Viral Hepatitis, STD, and TB Prevention, CDC.

Alcohol use during pregnancy is a major preventable cause of adverse alcohol-related outcomes, including birth defects and developmental disabilities.* Alcohol screening and brief intervention (ASBI) is an evidence-based primary care tool that has been shown to prevent or reduce alcohol consumption during pregnancy; interventions have resulted in an increase in the proportion of pregnant women reporting abstinence (odds ratio = 2.26; 95% CI = 1.43–3.56) (*1*). Previous national estimates have not characterized ASBI in populations of pregnant persons. Using 2017 and 2019 Behavioral Risk Factor Surveillance System (BRFSS) data, CDC examined prevalence of ASBI and characteristics of pregnant persons and nonpregnant women aged 18–49 years (reproductive-aged women) residing in jurisdictions that participated in the BRFSS ASBI module. During their most recent health care visit within the past 2 years, approximately 80% of pregnant persons reported being asked about their alcohol use; however, only 16% of pregnant persons who self-reported current drinking at the time of the survey (at least one alcoholic beverage in the past 30 days) were advised by a health care provider to quit drinking or reduce their alcohol use. Further, the prevalence of screening among pregnant persons who did not graduate from high school was lower than that among those who did graduate from high school or had at least some college education. This gap between screening and brief intervention, along with disparities in screening based on educational level, indicate missed opportunities to reduce alcohol use during pregnancy. Strategies to enhance ASBI during pregnancy include integrating screenings into electronic health records, increasing reimbursement for ASBI services, developing additional tools, including electronic ASBI, that can be implemented in a variety of settings (*2**,**3*).

There is no known safe amount of alcohol, type of alcohol, or timing of alcohol use during pregnancy or while trying to become pregnant. Alcohol use among pregnant persons remains a public health concern. During 2015–2017, 11.5% of pregnant U.S. women aged 18–44 years reported current drinking (*4*), and during 2018–2020, 13.5% of pregnant adults aged 18–49 years reported current drinking (*5*). Brief intervention or behavioral counseling conducted in a primary care setting has been shown to increase the likelihood of abstaining from alcohol during pregnancy (*1*). The U.S. Preventive Services Task Force recommends implementing ASBI for all adults aged ≥18 years in primary health care settings, including those who are pregnant, to reduce excessive alcohol use, which includes any alcohol use while pregnant (*6*). Despite these recommendations for universal screening, some populations might not be screened as frequently as others (*7*).

BRFSS is a cross-sectional, random-digit–dialed, annual telephone survey of noninstitutionalized U.S. adults aged ≥18 years^†^ that collects data on health-related behaviors. CDC analyzed data from 23 states and the District of Columbia^§^ that participated in an optional BRFSS ASBI module in 2017 and 2019^¶^ (unweighted sample size = 248,901; median response rate = 45.9% [2017] and 49.4% [2019]). For states that participated in the ASBI module both years (California, Kansas, and Nebraska), analytic weights were adjusted proportionally to their sample size for each year. Pregnant persons** and reproductive-aged women were compared by age, race and ethnicity,^††^ education level,^§§^ employment status,^¶¶^ disability status,*** HIV risk,^†††^ experience of frequent mental distress,^§§§^ chronic conditions,^¶¶¶^ health insurance status,**** having a usual health care provider,^††††^ residence in a state with expanded Medicaid,^§§§§^ cigarette use,^¶¶¶¶^ any alcohol use,***** and binge drinking.^†††††^ Analyses were conducted to estimate the prevalence of alcohol use and screening^§§§§§^ among pregnant persons and reproductive-aged women who visited a health care provider in the past 2 years. Prevalence of brief intervention^¶¶¶¶¶^ was calculated among pregnant persons.

Prevalence estimates and 95% CIs were standardized to the age distribution of persons who gave birth to a live singleton infant in 2017 using vital statistics data.****** Survey procedures with Taylor series variance and weights were used to account for the sample design and nonresponse. Wald chi-square tests were used to test for differences with p<0.05 considered statistically significant. All analyses were conducted using SAS (version 9.4; SAS Institute). BRFSS data are publicly available, and their use is not subject to human subjects review. This activity was reviewed by CDC and was conducted consistent with applicable federal law and CDC policy.^††††††^

Among 950 pregnant persons in jurisdictions included in the 2017 and 2019 BRFSS ASBI module, 13.3% reported current drinking and 6.9% reported binge drinking (Table 1). Among reproductive-aged women, 56.4% reported current drinking and 20.2% reported binge drinking. Overall, 80.1% of pregnant persons and 86.0% of reproductive-aged women reported being screened for alcohol use at their last visit to a health care provider (Table 2). Pregnant persons who did not graduate from high school reported a lower prevalence of alcohol screening (53.5%) compared with those who graduated from high school (83.4%) and those with at least some college education (84.5%). A higher proportion of pregnant persons who reported behaviors that might increase the risk for HIV transmission were screened (95.8%) than were those without reported risk behaviors (78.6%). No significant differences in screening prevalence among pregnant persons were observed based on race and ethnicity, disability status, frequent mental distress, health insurance status, having a usual health care provider, or living in a Medicaid expansion state. However, among reproductive-aged women, screening prevalence was lower among those who were non-Hispanic and of another race or ethnicity (i.e., American Indian or Alaska Native, Asian, Native Hawaiian or other Pacific Islander, or multiracial) than among those who were Hispanic or Latino, non-Hispanic Black or African American, and non-Hispanic White. Screening prevalence was also lower among reproductive-aged women who did not have health insurance than among those with any health insurance. Among pregnant persons who reported current drinking at the time of the survey, 96.7% (95% CI = 93.4–100.0) reported having been screened at their most recent health care visit.

**TABLE 1:** Age-standardized* characteristics of pregnant persons and nonpregnant reproductive-aged women — Behavioral Risk Factor Surveillance System, Alcohol Screening and Brief Intervention module, 23 states and the District of Columbia,^†^ 2017 and 2019

Characteristic^§^	Weighted % (95% CI)		P-value
	Pregnant persons^¶^ (unweighted n = 950)	Nonpregnant reproductive-aged women (unweighted n = 28,476)	
**Age group, yrs**			
18–24	25.1 (20.5–29.7)	22.4 (21.5–23.3)	<0.001
25–34	53.2 (48.0–58.4)	30.9 (30.0–31.8)	
35–49	21.7 (17.5–25.9)	46.7 (45.8–47.6)	
**Race and ethnicity**			
Black or African American, non-Hispanic	13.9 (10.1–17.7)	15.4 (14.5–16.3)	0.113
Hispanic or Latino	28.1 (23.4–32.8)	23.7 (22.7–24.7)	
White, non-Hispanic	45.7 (40.6–50.9)	48.6 (47.4–49.7)	
Other, non-Hispanic**	12.2 (8.4–16.1)	12.3 (11.4–13.1)	
**Education** ^††^			
Did not graduate from high school	15.3 (10.7–19.9)	11.0 (10.1–11.8)	0.116
Graduated from high school	24.1 (19.7–28.4)	23.9 (22.9–24.9)	
Some college or more	60.7 (55.4–66.0)	65.1 (63.9–66.3)	
**Employment status^§§^**			
Employed	57.3 (52.1–62.6)	62.3 (61.1–63.4)	0.030
Not employed	42.7 (37.4–47.9)	37.7 (36.6–38.9)	
**Disability status^¶¶^**			
Reported disability	13.7 (9.6–17.8)	18.5 (17.6–19.4)	0.016
No reported disability	86.3 (82.2–90.4)	81.5 (80.6–82.4)	
Reported behaviors that increase risk for HIV transmission***			
Yes	8.3 (5.7–11.0)	10.1 (9.4–10.9)	0.996
No	91.7 (89.0–94.3)	89.9 (89.1–90.6)	
**Mental distress^†††^**			
Frequent mental distress	11.6 (7.7–15.5)	16.9 (16.0–17.7)	0.030
No frequent mental distress	88.4 (84.5–92.3)	83.1 (82.3–84.0)	
**Chronic conditions^§§§^**			
Any chronic condition	55.4 (49.0–61.8)	57.1 (55.7–58.5)	0.123
No chronic condition	44.6 (38.2–51.0)	42.9 (41.5–44.3)	
**Health insurance status^¶¶¶^**			
Any health insurance	88.9 (85.7–92.2)	86.6 (85.8–87.5)	0.507
No health insurance	11.1 (7.8–14.3)	13.4 (12.5–14.2)	
Health care provider****			
Has a usual health care provider	75.2 (70.7–79.7)	76.5 (75.5–77.5)	0.033
Does not have a usual health care provider	24.8 (20.3–29.3)	23.5 (22.5–24.5)	
**Medicaid expansion^††††^**			
Lives in Medicaid expansion state	62.9 (58.0–67.7)	62.9 (62.1–63.7)	0.841
Does not live in Medicaid expansion state	37.1 (32.3–42.0)	37.1 (36.3–37.9)	
**Alcohol use**			
Current drinking**^§§§§^**	13.3 (8.9–17.6)	56.4 (55.2–57.5)	<0.001
Binge drinking^¶¶¶¶^	6.9 (3.0–10.8)	20.2 (19.2–21.1)	<0.001
Cigarette use*****			
Every day or some days	5.4 (2.7–8.0)	12.6 (11.8–13.3)	<0.001
No cigarette use	94.6 (92.0–97.3)	87.4 (86.7–88.2)	

**Abbreviation**: BRFSS = Behavioral Risk Factor Surveillance System.

* Prevalence estimates and 95% CIs were standardized to the age distribution of persons who gave birth to a live singleton infant in 2017 using vital statistics data. https://wonder.cdc.gov/

^†^ Alabama, Alaska, Arizona, Arkansas, California, Colorado, Connecticut, District of Columbia, Georgia, Illinois, Kansas, Maryland, Minnesota, Montana, Nebraska, Nevada, New Hampshire, North Carolina, Oklahoma, Rhode Island, South Carolina, Tennessee, Utah, and Wisconsin.

^§^ Not all response categories were mutually exclusive.

^¶^ Self-reported pregnancy was based on responses to the question, “To your knowledge, are you now pregnant?” This question is asked if the respondent’s sex is female and respondent was aged ≤49 years.

** Includes persons who are American Indian or Alaska Native, Asian, Native Hawaiian or other Pacific Islander, and multiracial.

^††^ Self-reported education level was based on computed levels as follows: “Did not graduate High School,” “Graduated High School,” “Attended College or Technical School,” and “Graduated from College or Technical School.” Responses to “Attended College or Technical School” and “Graduated from College or Technical School” were combined to a variable of “Some college or more.”

^§§^ Employment status included employed for wages or self-employed. Unemployment status included being out of work for ≥1 year, out of work for <1 year, a homemaker, a student, retired, or unable to work.

^¶¶^ Disability was defined as responding “yes” to any of the following questions: “Are you deaf or do you have serious difficulty hearing?,” “Are you blind or do you have serious difficulty seeing, even when wearing glasses?,” “Because of a physical, mental, or emotional condition, do you have serious difficulty concentrating, remembering, or making decisions?,” “Do you have serious difficulty walking or climbing stairs?,” “Do you have difficulty dressing or bathing?” and “Because of a physical, mental, or emotional condition, do you have difficulty doing errands alone such as visiting a doctor’s office or shopping?”

*** Respondents were classified as reporting behaviors that might increase the risk of HIV transmission if they reported at least one of the following: 1) injection of any drug other than prescribed in the past year, 2) being treated for a sexually transmitted disease in the past year, 3) having given or received money or drugs in exchange for sex in the past year, 4) had anal sex without a condom in the past year, or 5) had four or more sexual partners in the past year.

^†††^ Frequent mental distress was based on responses to the question, “Now thinking about your mental health, which includes stress, depression, and problems with emotions, for how many days during the past 30 days was your mental health not good?” where ≥14 days was considered frequent mental distress.

^§§§^ Chronic condition was defined as ever being told by a health care provider that the person had a heart attack, angina, coronary heart disease, stroke, hypertension (including gestational hypertension), diabetes (including gestational diabetes), arthritis, asthma, chronic obstructive pulmonary disease, emphysema, chronic bronchitis, depression, any cancer, or chronic kidney disease.

^¶¶¶^ Health insurance status was based on responses to the question, “Do you have any kind of health care coverage, including health insurance, prepaid plans such as health maintenance organizations, or government plans such as Medicare, or Indian Health Service?”

**** Having a usual health care provider was based on responses to the question, “Do you have one person you think of as your personal doctor or health care provider?” where one or more than one was included.

^††††^ States were included that had expanded Medicaid before 2017 or 2019, depending on the year or years each state was included in the BRFSS Alcohol Screening and Brief Intervention module survey. https://www.kff.org/medicaid/issue-brief/status-of-state-medicaid-expansion-decisions-interactive-map/

^§§§§^ Self-reported current drinking was based on the BRFSS calculated variable of “Adults who reported having had at least one drink of alcohol in the past 30 days.”

^¶¶¶¶^ Self-reported binge drinking was based on the BRFSS calculated variable of “Considering all types of alcoholic beverages, how many times during the past 30 days did you have ≥5 drinks [for men] or ≥4 drinks [for women] on an occasion?”

***** Cigarette use was based on responses to the questions, “Have you smoked at least 100 cigarettes in your entire life?” and “Do you now smoke cigarettes every day, some days, or not at all?” Responses of “every day” and “some days” were combined to create a dichotomous variable of cigarette use, and persons who responded “no” to the question “Have you smoked at least 100 cigarettes in your entire life?” were combined with persons who reported “not at all.”

**TABLE 2:** Age-standardized* prevalence of alcohol screening^†^ by a health care provider in the past 2 years, by pregnancy status among women of reproductive age — Behavioral Risk Factor Surveillance System, Alcohol Screening and Brief Intervention module, 23 states and the District of Columbia,^§^ 2017 and 2019

Characteristic^¶^	Alcohol screening prevalence			
	Pregnant persons** (unweighted n = 753*)		Nonpregnant reproductive-aged women (unweighted n = 22,440*)	
	Weighted % (95% CI)	P-value	Weighted % (95% CI)	P-value
**Total**	**80.1 (75.3–84.8)**	**—**	**86.0 (84.9–87.0)**	**—**
**Age group, yrs**				
18–24	78.8 (69.9–87.7)	0.738	83.0 (80.9–85.2)	<0.001
25–34	79.6 (72.8–86.4)		86.8 (85.3–88.3)	
35–49	83.4 (75.0–91.8)		87.3 (86.3–88.3)	
**Race and ethnicity**				
Black or African American, non-Hispanic	79.7 (67.1–92.3)	0.472	85.1 (82.9–87.3)	<0.001
Hispanic or Latino	79.0 (69.1–88.8)		86.3 (84.7–87.9)	
White, non-Hispanic	83.2 (77.2–89.2)		88.4 (87.3–89.6)	
Other, non-Hispanic^††^	69.6 (53.1–86.1)		77.1 (73.4–80.7)	
**Education^§§^**				
Did not graduate from high school	53.5 (35.5–71.5)	<0.001	82.4 (79.7–85.1)	<0.001
Graduated from high school	83.4 (75.1–91.7)		83.0 (81.2–84.9)	
Some college or more	84.5 (79.9–89.0)		87.8 (86.6–88.9)	
**Employment status^¶¶^**				
Employed	82.2 (76.6–87.9)	0.283	87.6 (86.5–88.8)	<0.001
Not employed	77.3 (69.4–85.2)		83.6 (81.9–85.2)	
Disability status***				
Reported disability	86.5 (78.1–94.9)	0.193	85.3 (83.6–87.1)	0.451
No reported disability	79.3 (74.1–84.5)		86.1 (85.0–87.3)	
**Reported behaviors that increase risk for HIV transmission** ^†††^				
Yes	95.8 (90.2–100.0)	<0.001	88.4 (85.2–91.7)	0.318
No	78.6 (73.5–83.7)		85.7 (84.7–86.8)	
**Mental distress^§§§^**				
Frequent mental distress	89.6 (81.7–97.5)	0.072	87.0 (85.1–88.9)	0.359
No frequent mental distress	79.4 (74.2–84.5)		85.8 (84.6–86.9)	
**Chronic conditions^¶¶¶^**				
Chronic condition	83.6 (76.8–90.4)	0.261	86.8 (85.5–88.2)	<0.001
No chronic condition	78.3 (69.7–87.0)		83.3 (81.4–85.3)	
Health insurance status****				
Any health insurance	80.4 (75.4–85.4)	0.672	87.0 (86.0–88.1)	<0.001
No health insurance	77.0 (63.2–90.9)		79.3 (76.4–82.1)	
**Health care provider^††††^**				
Has a usual health care provider	79.8 (74.3–85.3)	0.825	86.5 (85.4–87.7)	0.006
Does not have a usual health care provider	80.7 (71.6–89.7)		84.2 (82.2–86.2)	
**Medicaid expansion^§§§§^**				
Lives in Medicaid expansion state	78.8 (72.4–85.1)	0.498	85.7 (84.4–87.0)	0.317
Does not live in Medicaid expansion state	82.1 (75.6–88.6)		86.5 (85.2–87.7)	

**Abbreviation**: BRFSS = Behavioral Risk Factor Surveillance System.

* Prevalence estimates and 95% CIs were standardized to the age distribution of persons who gave birth to a live singleton infant in 2017 using vital statistics data. https://wonder.cdc.gov/

^†^ Alcohol screening was based on responses to the question, “You told me earlier that your last routine checkup was [within the past 2 years]. At that checkup, were you asked in person or on a form if you drink alcohol?” Among 950 pregnant persons who had a health checkup in the past 2 years, 753 (79.3%) had nonmissing data on alcohol screening. Among 28,476 nonpregnant women of reproductive age who had a health checkup in the past 2 years, 22,440 (78.8%) had nonmissing data on alcohol screening.

^§^ Alabama, Alaska, Arizona, Arkansas, California, Colorado, Connecticut, District of Columbia, Georgia, Illinois, Kansas, Maryland, Minnesota, Montana, Nebraska, Nevada, New Hampshire, North Carolina, Oklahoma, Rhode Island, South Carolina, Tennessee, Utah, and Wisconsin.

^¶^ Not all response categories were mutually exclusive.

** Self-reported pregnancy was based on responses to the question, “To your knowledge, are you now pregnant?” This question is asked if the respondent’s sex is female and respondent was aged ≤49 years.

^††^ Includes persons who are American Indian or Alaska Native, Asian, Native Hawaiian or other Pacific Islander, and multiracial.

^§§^ Self-reported education level was based on computed levels as follows: “Did not graduate High School,” “Graduated High School,” “Attended College or Technical School,” and “Graduated from College or Technical School.” Responses to “Attended College or Technical School” and “Graduated from College or Technical School” were combined to a variable of “Some college or more.”

^¶¶^ Employment status included employed for wages or self-employed. Unemployment status included being out of work for ≥1 year, out of work for <1 year, a homemaker, a student, retired, or unable to work.

*** Disability was defined as responding “yes” to any of the following questions: “Are you deaf or do you have serious difficulty hearing?,” “Are you blind or do you have serious difficulty seeing, even when wearing glasses?,” “Because of a physical, mental, or emotional condition, do you have serious difficulty concentrating, remembering, or making decisions?,” “Do you have serious difficulty walking or climbing stairs?,” “Do you have difficulty dressing or bathing?” and “Because of a physical, mental, or emotional condition, do you have difficulty doing errands alone such as visiting a doctor’s office or shopping?”

^†††^ Respondents were classified as reporting behaviors that might increase the risk for HIV transmission if they reported at least one of the following: 1) injection of any drug other than one prescribed in the past year, 2) being treated for a sexually transmitted disease in the past year, 3) having given or received money or drugs in exchange for sex in the past year, 4) had anal sex without a condom in the past year, or 5) had four or more sexual partners in the past year.

^§§§^ Frequent mental distress was based on responses to the question, “Now thinking about your mental health, which includes stress, depression, and problems with emotions, for how many days during the past 30 days was your mental health not good?” where ≥14 days was considered frequent mental distress.

^¶¶¶^ Chronic condition was defined as ever being told by a health care provider that the person had a heart attack, angina, coronary heart disease, stroke, hypertension (including gestational hypertension), diabetes (including gestational diabetes), arthritis, asthma, chronic obstructive pulmonary disease, emphysema, chronic bronchitis, depression, any cancer, or chronic kidney disease.

**** Health insurance status was based on responses to the question, “Do you have any kind of health care coverage, including health insurance, prepaid plans such as HMOs, or government plans such as Medicare, or Indian Health Service?”

^††††^ Having a usual health care provider was based on responses to the question, “Do you have one person you think of as your personal doctor or health care provider?” where one or more than one was included.

^§§§§^ States were included that had expanded Medicaid before 2017 or 2019, depending on the year or years each state was included in the BRFSS Alcohol Screening and Brief Intervention module survey. https://www.kff.org/medicaid/issue-brief/status-of-state-medicaid-expansion-decisions-interactive-map/

Approximately one quarter (25.3%; 95% CI = 19.6–31.0) of pregnant persons who received alcohol screening were offered advice from a health care provider about what level of drinking is harmful or risky to their health (including any amount of drinking during pregnancy), and 12.3% (95% CI = 7.6–17.0) were advised to reduce their intake or quit drinking (Figure). Among pregnant persons who reported being screened during their last health care visit and self-reported current drinking, 28.8% (95% CI = 12.2–45.4) were offered advice about what level of drinking is harmful or risky to health and 16.1% (95% CI = 6.9–25.3) were advised to reduce their alcohol intake or quit drinking.

**FIGURE:**
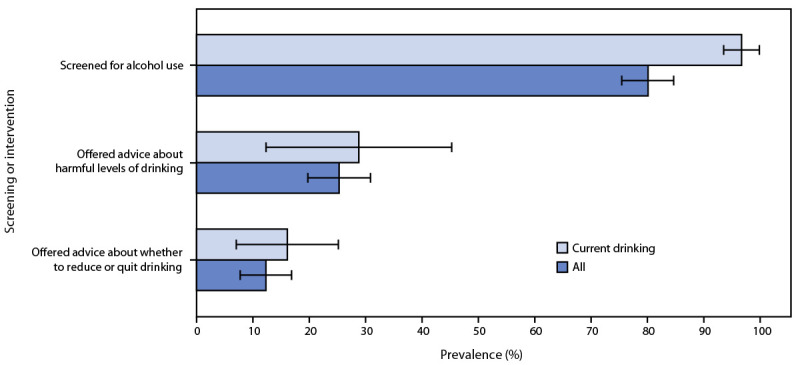
Prevalence* of age-standardized alcohol screening and brief intervention^†^ among pregnant persons — Behavioral Risk Factor Surveillance System, Alcohol Screening and Brief Intervention module, 23 states and the District of Columbia, 2017 and 2019^§^ **Abbreviation**: BRFSS = Behavioral Risk Factor Surveillance System. * With 95% CIs indicated by error bars. ^†^ Brief intervention was based on responses to the questions, “Were you offered advice about what level of drinking is harmful or risky for your health?” and “At your last routine checkup, were you advised to reduce or quit your drinking?” These questions are only asked if participants responded “Yes” to the question, “You told me earlier that your last routine checkup was [within the past 2 years]. At that checkup, were you asked in person or on a form if you drink alcohol?” Because of survey design, it could not be determined whether the health care provider screened for alcohol use and gave a brief intervention before or after the patient reported alcohol use, or if the patient was using alcohol at the time of the health care visit. Self-reported current drinking was based on the BRFSS calculated variable of “Adults who reported having had at least one drink of alcohol in the past 30 days.” ^§^ Alabama, Alaska, Arizona, Arkansas, California, Colorado, Connecticut, District of Columbia, Georgia, Illinois, Kansas, Maryland, Minnesota, Montana, Nebraska, Nevada, New Hampshire, North Carolina, Oklahoma, Rhode Island, South Carolina, Tennessee, Utah, and Wisconsin.

## Discussion

Despite recommendations for universal alcohol screening, approximately 20% of pregnant persons were not screened for alcohol use at their last visit to a primary health care provider, and among those with past 30-day alcohol use, only 16% who were screened were advised by a health care provider to quit drinking or reduce their alcohol use. Some groups of pregnant persons, such as those who did not graduate from high school and those who did not report behaviors that might increase the risk for HIV transmission, reported lower prevalences of screening compared with those who graduated from high school and those who reported behaviors that might increase HIV transmission risk. Screening prevalence was significantly lower among reproductive-aged women who did not have health insurance than among those with any health insurance, indicating that lack of health insurance might interfere with engaging in routine alcohol screening and subsequent interventions. In addition, racial and ethnic disparities in ASBI were observed among reproductive-aged women.

The American College of Obstetricians and Gynecologists recommends that health care providers conduct a brief intervention with all persons who are pregnant if they report any alcohol use (*8*). Approximately one third of pregnant persons who reported being screened during their most recent health care visit and self-reported current drinking received advice about what level of drinking is risky or harmful to health. This represents a missed opportunity for providers to discuss the potential adverse effects of alcohol consumption during pregnancy. Brief interventions can vary in length, can be delivered in a wide variety of health care settings, and can be delivered either in person or electronically.^§§§§§§^

The findings in this report are subject to at least six limitations. First, BRFSS relies on self-reported responses, which are subject to recall and social desirability biases. Second, not all pregnancies might be recognized at the time of health care visit or survey. Third, BRFSS does not ask for trimester of pregnancy, and although it is recognized that alcohol use varies across pregnancy (*9*), brief intervention is warranted irrespective of the timing of alcohol use during pregnancy. Fourth, because of the survey design, it could not be ascertained whether the health care provider screened for alcohol use and gave a brief intervention before or after the patient reported alcohol use, or if the patient was using alcohol at the time of the clinic visit. Fifth, specific sociodemographic subgroups of interest (e.g., veterans and sexual and gender minority groups) were not evaluated because of small sample sizes. Finally, because only jurisdictions that participated in the ASBI module were included, the findings in this report might not be generalizable to other jurisdictions.

Despite evidence that ASBI is effective in reducing alcohol use (*1*), this analysis indicates that ASBI is underutilized in certain populations of pregnant persons. Although alcohol screening among pregnant persons was high, one in five were not screened. Health care providers face multiple barriers in conducting ASBI (*10*); strategies to address these include integrating screenings into electronic health records, increasing reimbursement for ASBI services, implementing electronic ASBI (*2*), and developing training and tools for conducting ASBI in both traditional and nontraditional settings (*3*). Disparities in brief intervention highlight opportunities for expanding communication with patients who report alcohol consumption during pregnancy about associated risks to prevent and reduce adverse alcohol-associated pregnancy outcomes.

**Summary**

**What is already known about this topic?**

Alcohol screening and brief intervention (ASBI) is an evidence-based tool to reduce alcohol consumption in adults, including pregnant persons.

**What is added by this report?**

In 2017 and 2019, during their most recent health care visit, 80% of pregnant persons reported being asked about their alcohol use; only 16% of those with past 30-day alcohol consumption were advised by a health care provider to quit or reduce their alcohol use. Disparities in alcohol screening were observed among pregnant persons with lower educational attainment.

**What are the implications for public health practice?**

Implementation of recommended ASBI among pregnant persons can help prevent alcohol use or reduce current drinking. Strategies to enhance ASBI include integrating screenings into electronic health records, increasing reimbursement for ASBI services, and development of additional tools including electronic ASBI.

## Footnotes

*
https://www.cdc.gov/ncbddd/fasd/alcohol-use.html

†
https://www.cdc.gov/brfss/

§ Alabama, Alaska, Arizona, Arkansas, California, Colorado, Connecticut, District of Columbia, Georgia, Illinois, Kansas, Maryland, Minnesota, Montana, Nebraska, Nevada, New Hampshire, North Carolina, Oklahoma, Rhode Island, South Carolina, Tennessee, Utah, and Wisconsin.

¶ https://www.cdc.gov/brfss/questionnaires/modules/category2017.htm; https://www.cdc.gov/brfss/questionnaires/modules/category2019.htm

** Self-reported pregnancy was based on responses to the question, “To your knowledge, are you now pregnant?” This question was asked if the respondent’s sex was female and the respondent was aged <49 years.

†† Race and ethnicity was defined as non-Hispanic Black or African American, Hispanic or Latino, non-Hispanic White, and Other (including non-Hispanic American Indian or Alaska Native, non-Hispanic Asian, non-Hispanic Native Hawaiian or other Pacific Islander, and non-Hispanic multiracial).

§§ Self-reported education level was based on computed levels as follows: “Did not graduate High School,” “Graduated High School,” “Attended College or Technical School,” and “Graduated from College or Technical School.” Responses to “Attended College or Technical School” and “Graduated from College or Technical School” were combined to a variable of “Some college or more.”

¶¶ Employment status included employed for wages or self-employed. Unemployment status included being out of work for ≥1 year, out of work for <1 year, a homemaker, a student, retired, or unable to work.

*** Disability was defined as an affirmative response to any of the following questions: “Are you deaf or have serious difficulty hearing?,” “Are you blind or have serious difficulty seeing, even when wearing glasses?,” “Because of a physical, mental, or emotional condition, do you have serious difficulty concentrating, remembering, or making decisions?,” “Do you have serious difficulty walking or climbing stairs?,” “Do you have difficulty dressing or bathing?,” and “Because of a physical, mental, or emotional condition, do you have difficulty doing errands alone such as visiting a doctor’s office or shopping?”

††† Respondents were classified as reporting behaviors that might increase the risk for HIV transmission if they reported at least one of the following: 1) injection of any drug other than one prescribed in the past year, 2) being treated for a sexually transmitted disease in the past year, 3) having given or received money or drugs in exchange for sex in the past year, 4) had anal sex without a condom in the past year, or 5) had four or more sexual partners in the past year.

§§§ Frequent mental distress was based on responses to the question, “Now thinking about your mental health, which includes stress, depression, and problems with emotions, for how many days during the past 30 days was your mental health not good?” where ≥14 days was considered frequent mental distress.

¶¶¶ Chronic condition was defined as ever having been told by a health care provider that the person had a heart attack, angina, coronary heart disease, stroke, hypertension (including gestational hypertension), diabetes (including gestational diabetes), arthritis, asthma, chronic obstructive pulmonary disease, emphysema, chronic bronchitis, depression, any cancer, or chronic kidney disease.

**** Health insurance status was based on responses to the question, “Do you have any kind of health care coverage, including health insurance, prepaid plans such as HMOs, or government plans such as Medicare, or Indian Health Service?”

†††† Having a usual health care provider was based on responses to the question, “Do you have one person you think of as your personal doctor or health care provider?” where one or more was included.

§§§§ States were included that had expanded Medicaid before 2017 or 2019, depending on the year or years each state was included in the BRFSS ASBI module survey. https://www.kff.org/medicaid/issue-brief/status-of-state-medicaid-expansion-decisions-interactive-map/

¶¶¶¶ Cigarette use was based on responses to the questions, “Have you smoked at least 100 cigarettes in your entire life?” and “Do you now smoke cigarettes every day, some days, or not at all?” Responses of “every day” and “some days” were combined to create a dichotomous variable of cigarette use, and persons who responded “no” to the question, “Have you smoked at least 100 cigarettes in your entire life?” were combined with persons who reported “not at all.”

***** Self-reported alcohol use was based on the BRFSS calculated variable of “Adults who reported having had at least one drink of alcohol in the past 30 days.”

††††† Self-reported binge drinking was based on the BRFSS calculated variable of “Considering all types of alcoholic beverages, how many times during the past 30 days did you have 5 or more drinks [for men] or 4 or more drinks [for women] on an occasion?”

§§§§§ Alcohol screening was based on responses to the question, “You told me earlier that your last routine checkup was [within the past 2 years]. At that checkup, were you asked in person or on a form if you drink alcohol?”

¶¶¶¶¶ Among participants who responded “yes” to the question “You told me earlier that your last routine checkup was [within the past 2 years]. At that checkup, were you asked in person or on a form if you drink alcohol?,” brief intervention was based on responses to the questions, “Were you offered advice about what level of drinking is harmful or risky for your health?” and “At your last routine checkup, were you advised to reduce or quit your drinking?”

******
https://wonder.cdc.gov/

†††††† 45 C.F.R. part 46, 21 C.F.R. part 56; 42 U.S.C. Sect. 241(d); 5 U.S.C. Sect. 552a; 44 U.S.C. Sect. 3501 et seq.

§§§§§§
https://www.thecommunityguide.org/findings/alcohol-excessive-consumption-electronic-screening-and-brief-interventions-e-sbi

## References

[1] O’Connor EA, Perdue LA, Senger CA, Screening and behavioral counseling interventions to reduce unhealthy alcohol use in adolescents and adults: updated evidence report and systematic review for the US Preventive Services Task Force. JAMA 2018;320:1910–28. 10.1001/jama.2018.1208630422198

[2] Ondersma SJ, Beatty JR, Svikis DS, Computer-delivered screening and brief intervention for alcohol use in pregnancy: a pilot randomized trial. Alcohol Clin Exp Res 2015;39:1219–26. 10.1111/acer.12747 26010235 PMC4490994

[3] Mulia N, Schmidt LA, Ye Y, Greenfield TK. Preventing disparities in alcohol screening and brief intervention: the need to move beyond primary care. Alcohol Clin Exp Res 2011;35:1557–60. 10.1111/j.1530-0277.2011.01501.x 21599711 PMC3684172

[4] Denny CH, Acero CS, Naimi TS, Kim SY. Consumption of alcohol beverages and binge drinking among pregnant women aged 18–44 years—United States, 2015–2017. MMWR Morb Mortal Wkly Rep 2019;68:365–8. 10.1016/j.amepre.2020.05.017 31022164 PMC6483284

[5] Gosdin LK, Deputy NP, Kim SY, Dang EP, Denny CH. Alcohol consumption and binge drinking during pregnancy among adults aged 18–49 years—United States, 2018–2020. MMWR Morb Mortal Wkly Rep 2022;71:10–3. 10.15585/mmwr.mm7101a2 34990444 PMC8735564

[6] Curry SJ, Krist AH, Owens DK, ; US Preventive Services Task Force. Screening and behavioral counseling interventions to reduce unhealthy alcohol use in adolescents and adults: US Preventive Services Task Force recommendation statement. JAMA 2018;320:1899–909. 10.1001/jama.2018.16789 30422199

[7] Patel E, Bandara S, Saloner B, Heterogeneity in prenatal substance use screening despite universal screening recommendations: findings from the Pregnancy Risk Assessment Monitoring System, 2016–2018. Am J Obstet Gynecol MFM 2021;3:100419. 10.1016/j.ajogmf.2021.100419 34116233

[8] The American College of Obstetricians and Gynecologists. Committee opinion no. 496: at-risk drinking and alcohol dependence: obstetric and gynecologic implications. Obstet Gynecol 2011;118:383–8. 10.1097/AOG.0b013e31822c9906 21775870

[9] Ethen MK, Ramadhani TA, Scheuerle AE, ; National Birth Defects Prevention Study. Alcohol consumption by women before and during pregnancy. Matern Child Health J 2009;13:274–85. 10.1007/s10995-008-0328-2 18317893 PMC6090563

[10] Dozet D, Burd L, Popova S. Screening for alcohol use in pregnancy: a review of current practices and perspectives. Int J Ment Health Addict 2021:1–20. 10.1007/s11469-021-00655-3 34580577 PMC8457028

